# Esophageal microbiota composition and outcome of esophageal cancer treatment: a systematic review

**DOI:** 10.1093/dote/doab076

**Published:** 2021-11-11

**Authors:** Victor D Plat, Tessel M van Rossen, Freek Daams, Nanne K de Boer, Tim G J de Meij, Andries E Budding, Christina M J E Vandenbroucke-Grauls, Donald L van der Peet

**Affiliations:** Department of Gastrointestinal Surgery, Amsterdam UMC, VU University Medical Center, Amsterdam, The Netherlands; Department of Medical Microbiology and Infection Control, Amsterdam Institute for Infection and Immunity, Amsterdam UMC, VU University Medical Center, Amsterdam, The Netherlands; Department of Gastrointestinal Surgery, Amsterdam UMC, VU University Medical Center, Amsterdam, The Netherlands; Department of Gastroenterology and Hepatology, Amsterdam Gastroenterology Endocrinology Metabolism (AGEM) Research Institute, Amsterdam UMC, VU University Medical Center Amsterdam, The Netherlands; Department of Pediatric Gastroenterology and Hepatology, Amsterdam UMC, VU University Medical Center, Amsterdam, The Netherlands; In Biome B.V., Amsterdam, The Netherlands; Department of Medical Microbiology and Infection Control, Amsterdam Institute for Infection and Immunity, Amsterdam UMC, VU University Medical Center, Amsterdam, The Netherlands; Department of Gastrointestinal Surgery, Amsterdam UMC, VU University Medical Center, Amsterdam, The Netherlands

**Keywords:** anastomotic leakage, complications, esophageal cancer surgery, microbiology, neoadjuvant chemoradiation, survival

## Abstract

*Background:* The role of esophageal microbiota in esophageal cancer treatment is gaining renewed interest, largely driven by novel DNA-based microbiota analysis techniques. The aim of this systematic review is to provide an overview of current literature on the possible association between esophageal microbiota and outcome of esophageal cancer treatment, including tumor response to (neo)adjuvant chemo(radio)therapy, short-term surgery-related complications, and long-term oncological outcome. *Methods:* A systematic review of literature was performed, bibliographic databases were searched and relevant articles were selected by two independent researchers. The Newcastle-Ottawa scale was used to estimate the quality of included studies. *Results:* The search yielded 1303 articles, after selection and cross-referencing, five articles were included for qualitative synthesis and four studies were considered of good quality. Two articles addressed tumor response to neoadjuvant chemotherapy and described a correlation between high intratumoral *Fusobacterium nucleatum* levels and a poor response. One study assessed surgery-related complications, in which no direct association between esophageal microbiota and occurrence of complications was observed. Three studies described a correlation between shortened survival and high levels of intratumoral *F. nucleatum*, a low abundance of Proteobacteria and high abundances of *Prevotella* and *Streptococcus* species. *Conclusions:* Current evidence points towards an association between esophageal microbiota and outcome of esophageal cancer treatment and justifies further research. Whether screening of the individual esophageal microbiota can be used to identify and select patients with a predisposition for adverse outcome needs to be further investigated. This could lead to the development of microbiota-based interventions to optimize esophageal microbiota composition, thereby improving outcome of patients with esophageal cancer.

## INTRODUCTION

The human body contains trillions of microbes. The totality of microorganisms and their collective genetic material present on the surfaces of the human body is called the human microbiome.^1^ The whole of the many different microbial species that are present in a specific anatomical niche is usually referred to as the ‘microbiota’, such as the ‘skin microbiota’ or the ‘esophageal microbiota’. The number of bacteria in the esophagus is estimated at 10^3^–10^4^ both aerobes and anaerobes, whereas the colon contains 10^11^ mainly anaerobic, bacteria.^2^

Several esophageal diseases have been associated with changes in the esophageal microbiota composition.^3^ In reflux esophagitis and Barrett’s esophagus, a decreased abundance of *Streptococcus* species and an increase of gram-negative bacteria have been observed.^4–6^ In esophageal adenocarcinoma, a reduction of overall microbial diversity, a decrease of Firmicutes, and an increase of Proteobacteria and *Lactobacillus fermentum* have been reported.^7^^,^^8^ Microbiota changes observed in squamous cell carcinoma (SCC) include a reduction of *Streptococcus* species and an increase of *Fusobacterium nucleatum*.^9^^,^^10^ Furthermore, some evidence suggests that the oral flora is comparable to inherent esophageal microbiota composition and points towards an association between oral microbiota and esophageal diseases.^11^ Next to this, gastric diseases, including gastritis, peptic ulcer disease, and different stages of gastric cancer, have been associated to changes in gastric microbiota composition.^12^^,^^13^

Besides these observations, surgery outcomes have also been linked to specific microbiota signatures. The management of locally advanced esophageal cancer currently consists of trimodality therapy: esophagectomy combined with neoadjuvant chemoradiotherapy. Anastomotic leakage remains one of the most feared complications, resulting in an increased risk of reoperation and delayed discharge, leading to a substantial increase in morbidity, and hospital costs.^14^^,^^15^ In addition, an increased in-hospital mortality and recurrence of disease have been reported.^16^^,^^17^ Over 70 years, microbes have been thought to impact anastomotic healing. This concept is now gaining renewed interest, largely driven by the advances in DNA-based microbiota analysis techniques applied in recent studies, which provide a deeper insight in microbiota composition compared to traditional culturing methods.^18–20^ Evidence for a role of the microbiota in the development of anastomotic leakage comes from both animal studies and clinical colorectal research and has increased the interest in potential effects of the esophageal microbiota on esophageal surgery outcome.^21–23^

In addition to the link between the microbiota composition and treatment complications, several studies have suggested a correlation between the microbiota and (long-term) disease outcome in esophageal cancer. Following trimodality therapy, ~35% of patients are diagnosed with tumor recurrence after a minimum follow-up of 24 months.^24^ In colorectal surgery, it has been demonstrated that cancer cells are detectable in the intestinal lumen after surgery, located on circular stapling devices and on suture lines at the anastomotic site.^25^ The same phenomenon can be assumed to occur in esophageal surgery. Specific alterations in the intestinal microbiota could possibly result in a microenvironment that promotes seeding of these exfoliated cancer cells. Whether these mechanisms truly facilitate cancer recurrence remains to be explored.

The aim of this systematic review is to provide a structured overview of the literature regarding the association between the esophageal microbiota and outcome after treatment for esophageal cancer. The study addresses (i) tumor response to (neo)adjuvant chemo(radio)therapy, (ii) short-term surgery-related complications (i.e. infectious complications and anastomotic leakage), and (iii) long-term oncological outcome (i.e. recurrence and survival). This guide future research and possibly result in development of targeted interventions to manipulate the esophageal microbiota composition and improve short- and long-term outcome in patients with esophageal cancer.

## METHODS

### Study strategy

A systematic review of peer-reviewed studies examining the association between the esophageal microbiota and outcome after esophageal cancer treatment was performed. This systematic review was reported according to the preferred reporting items for systematic reviews and meta-analysis (PRISMA) statement.^26^

### Search and selection of articles

A comprehensive literature search was conducted using the bibliographic databases PubMed and Embase from inception to 13 October 2020. The search terms included controlled terms (MesH in PubMed and Emtree in Embase) as well as free text terms. The keywords ‘esophagus’ and ‘microbiome’ were used as index terms or free-text words (including synonyms and closely related words). The search was performed without date, language, or publication status restriction. Duplicate articles were excluded. The full search strategies can be found in the supplemental information. Title and abstract of all identified citations were screened for eligibility by two independent reviewers (VP and TvR) using the following inclusion criteria: (i) original articles that analyzed the esophageal microbiota in patients or animals with esophageal or gastroesophageal junction cancer; (ii) evaluation of one of the following outcomes: (A) effect on (neo)adjuvant chemo(radio)therapy response, (B) effect on short-term surgery-related complications (i.e. anastomotic leakage and infectious complications), and (C) effect on long-term oncological outcome (i.e. recurrence and survival); and (iii) full-text availability in English. Systematic reviews were excluded after assessment of cross-references. Study protocols, letters and conference abstracts were excluded. Disagreements were handled by consensus. Full-texts of eligible citations were retrieved and individually assessed by two reviewers (VP and TvR). Data of these studies were systematically extracted in a predesigned table (VP) and confirmed by a second investigator (TvR). All articles meeting the eligibility criteria were included, reasons for exclusion were documented. Finally, reference lists of included studies were checked for additional studies.

### Quality assessment and risk of bias

The Newcastle-Ottawa scale for quality assessment of nonrandomized studies (NOS scale) was used to estimate the quality of included studies.^27^ This scale has previously been used in systematic reviews involving the gut microbiota ^28^ and assesses the research design, selection strategy, reliability of outcome determination, and follow-up adequacy. Depending on domain (selection, comparability, and outcome) a maximum of 2–4 stars was awarded. Based on the number of stars in each domain, the quality of the study was categorized as good, fair, or poor.^27^

## RESULTS

### Study selection and quality assessment

**Fig. 1:**
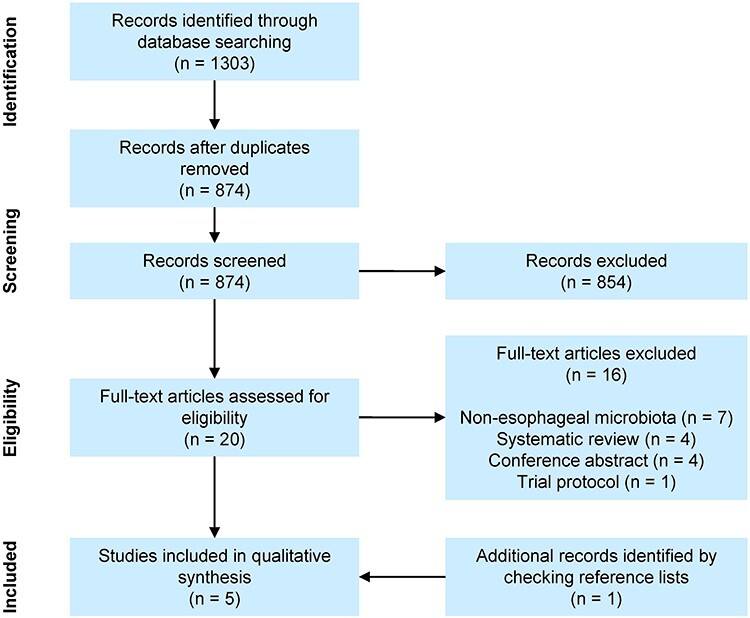
Flow diagram with schematic presentation of study selection and exclusion stages.

The search identified 1303 citations, after duplicate removal 874 citations were screened based on title and abstract. This resulted in 20 articles that were assessed for eligibility, of which 16 were excluded. One additional article was included by cross-reference checking, leading to a total of five articles included for qualitative synthesis (Fig. 1). One research group authored for three out of five articles. Two articles assessed the role of the esophageal microbiota in tumor response to neoadjuvant chemotherapy, one article focused on short-term surgery-related complications (i.e. infection and anastomotic leakage) and three articles studied the association between the esophageal microbiota and long-term oncological outcome (i.e. recurrence and survival; Table 1). Based on the NOS scale, four out of five studies were considered to be of good quality (Table 2).

**Table 1:** Summary of included articles

Neoadjuvant chemotherapy response								
Author	*N*	Cancer type	Treatment	Sample collection	Microbiota analysis	Outcome	Quality	Results
Yamamura†^*^ 2019 (10)	101	SCC	Neoadjuvant docetaxel, cisplatin and 5-FU	Intraoperative tumor specimen (FFPE)	*F. nucleatum* qPCR	RECIST, metabolic response and TRG	Good	High level of intratumoral *F. nucleatum* was an independent risk factor for poor tumor, metabolic and pathological response.
Liu† 2021 (29)	120	SCC	Neoadjuvant docetaxel, cisplatin and 5-FU	Intraoperative tumor specimen (FFPE)	*F. nucleatum* qPCR	Metabolic response and TRG	Good	Poor metabolic and pathological response in high *F. nucleatum* group.
Short-term surgery-related complications								
Author	*N*	Cancer type	Treatment	Sample collection	Microbiota analysis	Outcome		Results
Reddy 2018 (32)	55	EAC 80%, SCC 13% and benign 7%	nCRT 80% and transhiatal esophagectomy	Intraoperative esophageal mucosal biopsy	16S rRNA PCR	Anastomotic leakage and pneumonia	Poor	In esophageal/gastric samples *Akkermansia, Lactobacillus*, *Escherichia/Shigella* were dominant genera. No direct correlation between esophageal microbiota patterns and complications.
Long-term oncological outcome								
Author	*N*	Cancer type	Treatment	Sample collection	Microbiota analysis	Outcome		Results
Yamamura† 2016 (33)	325	EAC 4%, SCC 92% and other 4%	nCRT 14%, NAC 22% and esophagectomy	Intraoperative tumor specimen (FFPE)	*F. nucleatum* qPCR	Cancer-specific and overall survival (FU 2.5 to 11 years)	Good	In patients with intratumoral *F. nucleatum* expression a significantly shorter cancer-specific and overall survival was reported.
Liu 2018 (34)	45	SCC	Esophagectomy	Intraoperative tumor tissue resection	16S rRNA PCR	Overall survival (FU 1 to 2.5 years)	Good	Significant association between low abundance of Proteobacteria and shortened survival. A high abundance of *Prevotella* and *Streptococcus* correlated with shortened survival.
Yamamura†^*^ 2019 (10)	551	SCC	NAC or nCRT 47% and esophagectomy	Intraoperative tumor specimen (fresh frozen tumor tissue and FFPE)	*F. nucleatum* qPCR	Recurrence rate and recurrence-free survival (FU between 20 and 32 months)	Good	A significant association between high levels of intratumoral *F. nucleatum* and advanced disease, recurrence rates and shortened recurrence-free survival was reported.

5-FU indicates 5-fluorouracil; EAC, esophageal adenocarcinoma; *F. nucleatum*, *Fusobacterium nucleatum*; FFPE, formalin-fixed paraffin-embedded; FU, follow-up; NAC, neoadjuvant chemotherapy; nCRT, neoadjuvant chemoradiotherapy; PCR, polymerase chain reaction; qPCR, quantitative polymerase chain reaction; RECIST, response evaluation criteria in solid tumors; rRNA, ribosomal RNA; SCC, squamous cell carcinoma; TRG, tumor regression grade. †Same research group. ^*^Same article; this study comprised a total of 551 cases with SCC, of which 101 received neoadjuvant chemotherapy.

**Table 2:** Quality assessment of included studies using the NOS scale

	Selection (4 domains, max 4 stars)				Comparability (2 domains, max 2 stars)		Outcome (3 domains, max 3 stars)			
Study	Representativeness of exposed cohort	Selection of non-exposed cohort	Ascertainment of exposure	Outcome of interest not present at start	Primary microbiota analysis	Adjusted for confounding	Assessment of outcome	Follow-up long enough	Follow-up adequate	Overall quality score
Yamamura 2016 (33)	^*^	^*^	^*^	^*^	^*^	^*^	^*^	^*^	−	8/9
Reddy 2018 (32)	−	^*^	^*^	^*^	−	−	^*^	^*^	−	5/9
Liu 2018 (34)	^*^	^*^	^*^	^*^	^*^	^*^	^*^	^*^	−	8/9
Yamamura 2019 (10)	^*^	^*^	^*^	^*^	^*^	^*^	^*^	^*^	−	8/9
Liu 2021 (29)	^*^	^*^	^*^	^*^	^*^	−	^*^	^*^	−	7/9

The ^*^ indicate stars.

### Neoadjuvant chemotherapy response

Two articles, conducted by the same research group, with different first authors, examined the association between *F. nucleatum* levels and neoadjuvant chemotherapy response.^10^^,^^29^ The authors describe that the presence of *F. nucleatum* (a gram-negative and anaerobic bacterium) in the intestine has been associated with poor response in colorectal cancer, and therefore their aim was to study the possible link between *F. nucleatum* levels and chemotherapy response in esophageal cancer.^30^ The study of Yamamura *et al.* included a cohort of 101 patients who received neoadjuvant chemotherapy; the study population of Liu *et al.* consisted of 120 patients. All patients had SCC and were included in the same time period, but it is unclear if there was any overlap between study populations. All patients were treated with cisplatin and 5-fluorouracil either with or without docetaxel and subsequently underwent esophagectomy. Formalin-fixed paraffin-embedded (FFPE) tumor tissues were collected intraoperatively and *F. nucleatum* levels were measured by quantitative real-time PCR (qPCR). No routine perioperative antibiotic therapy was described in the studies.

Yamamura *et al.* first determined the *F. nucleatum* level cut-off threshold that provided the highest sensitivity and specificity to predict SCC recurrence in the training cohort (*n* = 207) and thereby categorized tumors into a ‘high’ and ‘low’ *F. nucleatum* group. Subsequently, chemotherapy response was compared between ‘high’ and ‘low’ *F. nucleatum* groups in the validation cohort (*n* = 101). Liu *et al.* detected *F. nucleatum* in 35/120 cases and compared outcome between these *F. nucleatum* ‘positive’ or ‘negative’ groups. Both studies assessed chemotherapy response using metabolic response rates and histopathological response. Metabolic response was defined by the maximum standardized uptake value (SUVmax) of 18F-fluoro-deoxy-glucose (FDG) in the target lesion. Metabolic responders were defined as >30% reduction in SUVmax or FDG uptake. Histopathological response was classified by tumor regression grade (TRG), responders were defined as grade 1–3 (<50% viable tumor cells). In addition, Yamamura *et al.* evaluated chemotherapy response using response evaluations criteria in solid tumors (RECIST) version 1.1, by measuring decrease in tumor volume on CT imaging.^31^ Responders were defined as >30% decrease in the sum of diameters of all target lesions.

Yamamura *et al.* observed that the high *F. nucleatum* group had a lower number of chemotherapy responders according to the RECIST (42.9% [12/28]) compared to the low *F. nucleatum* group (67.1% [49/73], *P* = 0.04). Patients with high *F. nucleatum* levels also had lower metabolic and histopathological response rates compared to patients with low *F. nucleatum* levels (47.6% vs. 87.7%, *P* = 0.0006 and 3.6% vs. 30.1%, *P* = 0.003, respectively). Similar findings were observed by Liu *et al.*: significantly less patients with high *F. nucleatum* levels showed a metabolic response (37.0% vs. 88.2%, *P* < 0.001) and histopathological response (2.9% vs. 25.9%, *P* < 0.001) compared to patients with low *F. nucleatum* levels. Furthermore, *F. nucleatum* levels in 30 esophageal biopsy specimens collected before treatment were significantly lower in chemotherapy responders than in nonresponders (*P* = 0.016). The authors suggest that these results could imply a causal relationship between the presence and abundance of *F. nucleatum* and response to chemotherapy and emphasize its potential as a pretreatment response predictor and possible target for antibiotic therapy.

### Short-term surgery-related complications

To date, the association between the perioperative esophageal microbiota composition and the incidence of complications following esophagectomy was investigated in one study.^32^ A total of 55 patients underwent transhiatal esophagectomy for adenocarcinoma (*n* = 44), SCC (*n* = 7), or benign disease (*n* = 4). Perioperative antibiotic therapy (cefazolin or vancomycin, dosage specifics were not available) was routinely administered. Esophageal and gastric mucosal samples were collected intraoperatively and analyzed by 16S rRNA PCR sequencing. In addition, saliva samples were collected pre- and postoperatively. The occurrence of surgical complications, including anastomotic leakage and pneumonia, was documented prospectively. Leakage was diagnosed using barium esophagogram or by clinical deterioration requiring opening of the neck wound. Pneumonia was defined by shortness of breath, leukocytosis, changes on X-ray imaging, and required antibiotics. In case of anastomotic leakage or pneumonia, neck wound swabs or sputum samples were collected, respectively. The dominant genera detected in the esophageal and gastric samples were *Akkermansia* species*, Lactobacillus* species and *Escherichia/Shigella* species. No differences in microbiota composition were observed between histological tumor types or stages. Anastomotic leakage and pneumonia occurred in 10 (18%) and 2 (3.7%) patients, respectively. The authors report a significant difference in microbiota composition between preoperative saliva samples and intraoperative gastric mucosa samples in patients who developed anastomotic leakage (*P* = 0.015), assessed by principal coordinate analysis. No direct correlation between esophageal microbiota patterns and anastomotic leakage was described. Furthermore, no differences were observed in microbiota profiles between patients with or without postoperative pneumonia.

### Long-term oncological outcome

Three studies addressed the association between the esophageal microbiota and long-term outcome following esophagectomy.^10^^,^^33^^,^^34^ Two of these studies, both by Yamamura *et al.*, have included patients in the same time period, however it is unclear if study populations overlap.^10^^,^^33^ The latter also evaluated neoadjuvant chemotherapy response and was previously described in the paragraph ‘Chemotherapy response’.^8^ The third study was performed by Liu *et al.* and published in 2018.^34^

Both studies of Yamamura *et al.* assessed the association between *F. nucleatum* and oncological outcome.^10^^,^^33^ The study of Yamamura *et al.* (2016) included 325 patients with SCC (*n* = 300), EAC (*n* = 12), or another diagnosis (*n* = 13); the study of Yamamura *et al.* (2019) comprised 551 SCC patients, divided into a training cohort (*n* = 207) and a validation cohort (*n* = 344). All patients underwent esophagectomy and FFPE tumor tissues were collected intraoperatively. *Fusobacterium nucleatum* levels were measured by qPCR. In the 2016 study of Yamamura *et al.*, patients were divided into two groups based on intratumoral *F. nucleatum* detection; the *F. nucleatum* positive group consisted of 74 patients (23%). In the 2019 study, as described in the paragraph ‘Chemotherapy response’, tumors were categorized into a ‘high’ and ‘low’ *F. nucleatum* group based on SCC recurrence in the training cohort. Subsequently, outcomes were compared between the positive and negative *F. nucleatum* groups (Yamamura *et al.* 2016), and between the high and low *F. nucleatum* groups (Yamamura *et al.* 2019). Long-term oncological outcomes of interest were cancer-specific survival, overall survival, recurrence, and recurrence-free survival.^10^^,^^33^

Yamamura *et al.* (2016) found that patients with a positive qPCR for intratumoral *F. nucleatum* had a significantly shorter cancer-specific survival (log-rank *P* = 0.0039) and overall survival (log-rank *P* = 0.046) compared to patients in the *F. nucleatum* negative group. Also, in multivariate Cox regression analysis adjusted for clinical, pathologic, and epidemiologic features, *F. nucleatum* positive patients had a significantly higher risk of cancer-related mortality than *F. nucleatum* negative patients (HR = 1.78, 95% CI 1.06–2.94, *P* = 0.032). In the study published in 2019, *F. nucleatum* levels were significantly higher in patients with tumor recurrence compared to patients without recurrence (training cohort: *P* = 0.04 and validation cohort: *P* = 0.01). Furthermore, patients with high *F. nucleatum* levels had a significantly shorter recurrence-free survival compared to patients with low *F. nucleatum* levels (log rank test, training cohort: *P* = 0.02 and validation cohort: *P* = 0.003). In a multivariate Cox regression analysis adjusted for several clinical and pathological features, high *F. nucleatum* levels remained an independent risk factor for a shorter recurrence-free survival (training cohort: HR = 1.72, 95% CI 1.12–2.70, *P* = 0.01, validation cohort: HR = 1.70, 95% CI 1.06–2.65, *P* = 0.03). When patients were stratified by clinical tumor stage (cT1 and cT2–4), patients with early stage (cT1) and high *F. nucleatum* levels had a shorter recurrence-free survival than cT1 patients with low *F. nucleatum* levels*,* and survival was similar to that of patients with advanced stage of disease (cT2–4).

The third study on the association between the esophageal microbiota composition and long-term oncological outcome following esophagectomy was performed by Liu *et al.*^34^ The authors prospectively studied 45 patients who underwent esophagectomy without neoadjuvant therapy. Patients who used antibiotics within two months prior to surgery were excluded. Tumor tissue samples were obtained intraoperatively and the esophageal microbiota was analyzed by 16S rRNA sequencing. At the phylum level, patients with a low abundance of Proteobacteria had a shorter survival than patients with a high abundance of Proteobacteria (*P* = 0.013). In patients with a high abundance of *Prevotella* and *Streptococcus,* a significantly lower survival was observed when compared to patients with low abundances of these genera (*P* = 0.015 and *P* = 0.006, respectively). Also, in multivariate Cox regression analysis the combination of high *Streptococcus* and *Prevotella* abundances was found to be an independent predictor of survival (HR: 6.094, 95% CI 1.072–34.646, *P* = 0.042).

## DISCUSSION

This systematic literature review describes the limited available data on the association between esophageal microbiota composition and treatment outcome in esophageal cancer. Only five studies assessed this association, four of these five studies were considered of good quality and three of these studies were conducted by the same research group. Overlap of study populations could not be ruled out. These few studies, however, point towards a possible association between esophageal microbiota composition and treatment outcome. Considering chemotherapy response, two studies found a potential association between high intratumoral *F. nucleatum* levels and a lower response rate to neoadjuvant chemotherapy. Only one small study focused on surgical complications, but no association between the esophageal microbiota and anastomotic leakage or pneumonia could be identified. Considering long-term oncological outcome, high levels of *F. nucleatum*, a low abundance of Proteobacteria and high abundances of *Prevotella* and *Streptococcus* appeared to correlate with a shortened survival. Current evidence is limited, yet these interesting results justify further research to elucidate the role of esophageal microbiota in the treatment of esophageal cancer.

The mechanisms by which *F. nucleatum* might influence chemoresistance in SCC cell lines were examined by Yamamura *et al.* (2019) using *in vitro* co-cultured assays.^10^ Transmission electron microscopy and laser scanning confocal microscopy were used to visualize the ability of *F. nucleatum* to invade SCC cells and to survive as an intracellular pathogen. *Fusobacterium nucleatum* induced chemotherapy resistance was suggested by the finding that addition of docetaxel, cisplatin and 5-fluorouracil to SCC cell lines cultured in the presence of *F. nucleatum* resulted in a significantly higher SCC cell proliferation compared to when these chemotherapeutic agents were added to SCC cells without *F. nucleatum*. Furthermore, the authors detected the upregulation of multiple autophagy-inducing or -promoting proteins (LC3, LC3B-II, ATG7, and Beclin-1) in *F. nucleatum* co-cultured SCC cell lines. Since the chemotherapy resistance by *F. nucleatum* could be reversed by inhibiting autophagy, the authors concluded that autophagy plays a pivotal role in *F. nucleatum* induced chemoresistance. In human colorectal cancer cell lines, similar autophagy promoting mechanisms, similar signaling proteins (ATG7, LC3, and LC3-II), and a similar resistance to Oxaliplatin and 5-FU was observed.^35^

Only one study addressed short-term complications of esophageal cancer surgery, in which no direct association between esophageal microbiota and complications was observed. A small sample size (*n* = 55, of which 10 patients developed anastomotic leakage and only two patients developed pneumonia) and heterogeneous population (EAC, SCC, and benign diseases) might have contributed to this negative result. The lack of more studies with negative findings in this respect, may of course be the result of publication bias. In contrast, some interesting findings have been observed in colorectal cancer research: in perioperatively collected colorectal mucosal biopsies, a low microbial diversity and relative enrichment of *Lachnospiraceae* and *Bacteroidaceae* were associated with a higher risk of developing anastomotic leakage.^23^ A proposed underlying mechanism of this finding is that the lack of diversity could facilitate the overgrowth of pathogenic bacteria, leading to anastomotic leakage.

How the esophageal microbiota might influence long-term oncological outcome remains to be elucidated. Yamamura *et al.* (2016) observed an enrichment of the ‘cytokine-cytokine reception interaction pathway’ in esophageal cancer tissues, in which CCL20 was the most upregulated chemokine. CCL20 expression was significantly higher in *F. nucleatum* positive tumor tissues compared to *F. nucleatum* negative tissues. The authors emphasize the potential of *F. nucleatum* as a prognostic biomarker, and propose that it may be possible to modulate it by antibiotic therapy, to improve outcome after esophagectomy.

Several forms of bias might have influenced the results of the included studies. First, sample sizes were small. Next, three out of five studies only assessed one bacterial species, *F. nucleatum*, instead of the complete esophageal microbiota, which consists of at least 150 different species.^4^^,^^36^ Although pathophysiological studies have shown the relevance of this species, the association between *F. nucleatum* levels and a worse chemotherapy response and survival might hypothetically be secondary to other, more relevant changes in the esophageal microbiota composition. Finally, several factors can influence the esophageal microbiota composition, such as age,^6^ tobacco use,^37^ medication,^38^ acid suppression therapies,^39^ and diet.^40^^,^^41^ These factors should be considered when interpreting microbiota data and corrected for when necessary. Most of these factors were not reported in the included studies. Although Liu *et al.* excluded patients with antibiotic use in the 2 months prior to surgery and Reddy *et al*. reported that all patients received cefazolin or vancomycin preoperatively, the other research groups did not provide data on antibiotic use. Moreover, no correction for potential (other) microbiota-influencing factors was performed in any of the studies.

Another challenge in esophageal microbiota studies is to obtain samples that provide a true representation of the esophageal microbiota. In order to use the microbiota as predictive or diagnostic marker of disease (course), sample collection should also be practical and patient friendly. All studies described in this review collected esophageal tissue intraoperatively, which probably accurately represents the esophageal microbiota composition, since this circumvents the risk of contamination of samples obtained by endoscopy, but obviously is highly invasive.^42^ Alternatives are endoscopic and non-endoscopic sampling methods, but pose risk of contamination from the oral cavity, which is characterized by a much higher bacterial concentration than the esophagus.^7^^,^^43^ Besides the sampling method, the preservation conditions of tissues can highly influence the results of microbiota studies. In three of the included studies, *F. nucleatum* qPCR was performed on FFPE samples.^10^^,^^29^^,^^33^ This preservation method might not impact the detection of single bacterial species, but can influence the results when analyzing entire microbiota profiles.^44^

Two different analysis techniques were used in the reviewed studies: qPCR and 16S rRNA sequencing. Yamamura (2016 and 2019) en Liu (2021) used specific qPCR to detect *F. nucleatum* levels in tumor tissues.^10^^,^^29^^,^^33^ This method targets a beforehand defined bacterial species and measures the absolute amount of DNA of this species in a sample. A limitation of this approach is that only *F. nucleatum* is detected, instead of the complete esophageal microbiota. Reddy and Liu (2018) used 16S sequencing for microbiota profiling.^32^^,^^34^ This technique is based on bacterial species-specific differences in the sequence of 16S ribosomal rDNA. Since all bacteria contain at least one rDNA allele, it is possible to obtain a profile of the entire microbiota and compare relative abundances of bacterial species within a sample. The combination of 16S/next generation sequencing (eventually supplemented by metagenomics) and detection of *F. nucleatum* levels could provide a more complete picture of the esophageal microbiota, and might help to identify specific microbial markers associated with treatment response. These results may progress our knowledge of pathogenesis of esophageal cancer and response to treatment.

The next step could be to modify the microbiota in a personalized matter by (a combination of) targeted antibiotics, probiotics, or prebiotics. Small spectrum antibiotics can eliminate or suppress undesirable microbes, probiotics may introduce missing microbial components and prebiotics are functional food ingredients that might change the composition and/or activity of the gastrointestinal microbiota by stimulate the proliferation of beneficial microbes. Longitudinal assessment of preoperative fecal microbiota composition in esophageal cancer patients has led to the discovery of significant microbial changes due to neo-adjuvant cisplatin, docetaxel and 5-FU. The administration of synbiotics, comprising a combination of probiotics and prebiotics, during neoadjuvant chemotherapy cycles might reduce these effects by restoring the intestinal microbiota composition, without significantly influencing the clinical response rate.^45^ Considering postoperative complications, one study assessed the effect of reducing the numbers of Proteobacteria in the intestinal microbiota by the perioperative administration of specific nonabsorbable antibiotics, but this did not affect the frequency of anastomotic leakage.^46^ Tanaka *et al.* observed that the perioperative use of synbiotics led to a favorable balance of fecal microbiota, but that did not result in a significant reduction of infectious complications.^47^

In conclusion, evidence regarding the association between the esophageal microbiota and treatment outcome of esophageal cancer is limited. Whether screening of the individual esophageal microbiota can be used to identify patients with a predisposition for incomplete chemotherapy response, postoperative complications or cancer recurrence remains to be clarified. Standardization of sampling, storage conditions, and analysis is necessary for a reliable comparison of study outcomes and to draw firm conclusions.
